# Supplementary material for: NINJ1 mediates inflammatory cell death, PANoptosis, and lethality during infection conditions and heat stress
Source: Nat Commun. 2024 Feb 26;15:1739. doi: 10.1038/s41467-024-45466-x (PMC10897308; doi:10.1038/s41467-024-45466-x)
Supplement: Supplementary file 1 — Supplementary Information [file 41467_2024_45466_MOESM1_ESM.pdf]

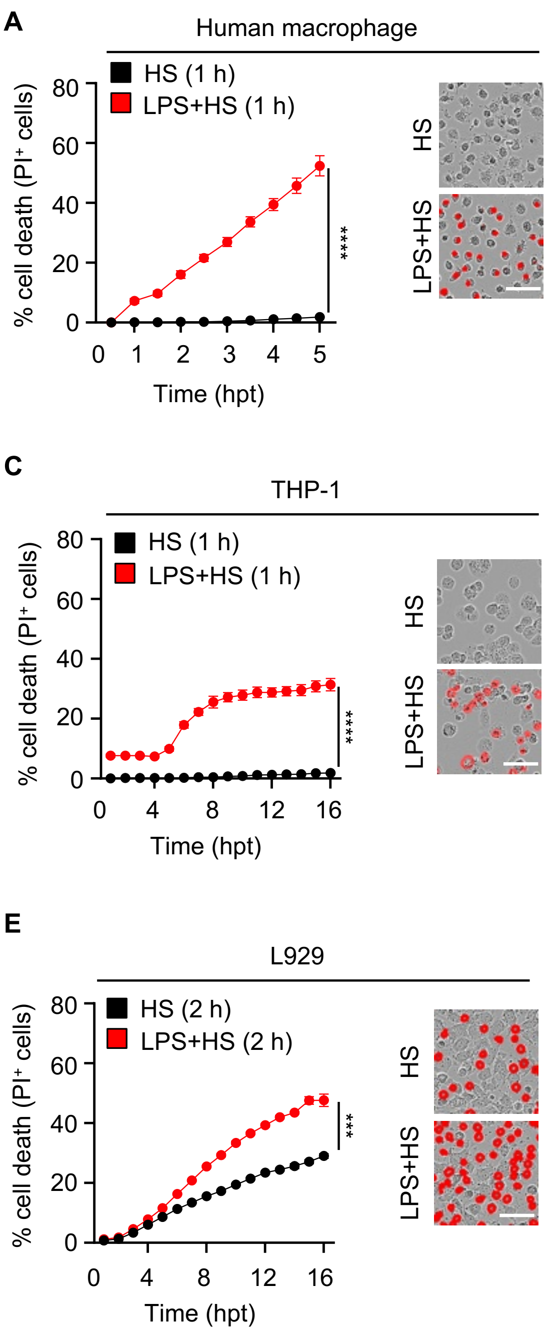

Supplementary Fig. 1. LPS priming potentiates heat stress-induced cell death in human and mouse cells

A–E Real-time analysis of cell death in human macrophages (A), human monocytes (B), THP-1 cells (C), RAW264.7 cells (D), and L929 cells (E) with and without LPS priming in response to heat stress (HS) for the indicated time. Representative images of cell death are shown in human macrophages at 5 h post-treatment (hpt) (A), human monocytes at 5 hpt (B), THP-1 cells at 16 hpt (C), RAW264.7 cells at 5 hpt (D), and L929 cells at 16 hpt (E). **A–E** Data are shown as mean ± SEM; ****P* < 0.001 and *****P* < 0.0001 (two-tailed *t*-test; n = 4 from 4 biologically independent samples). Images are representative of at least three independent experiments. Scale bar, 50 µm. Exact *P* values are presented in Supplementary Data file 2.

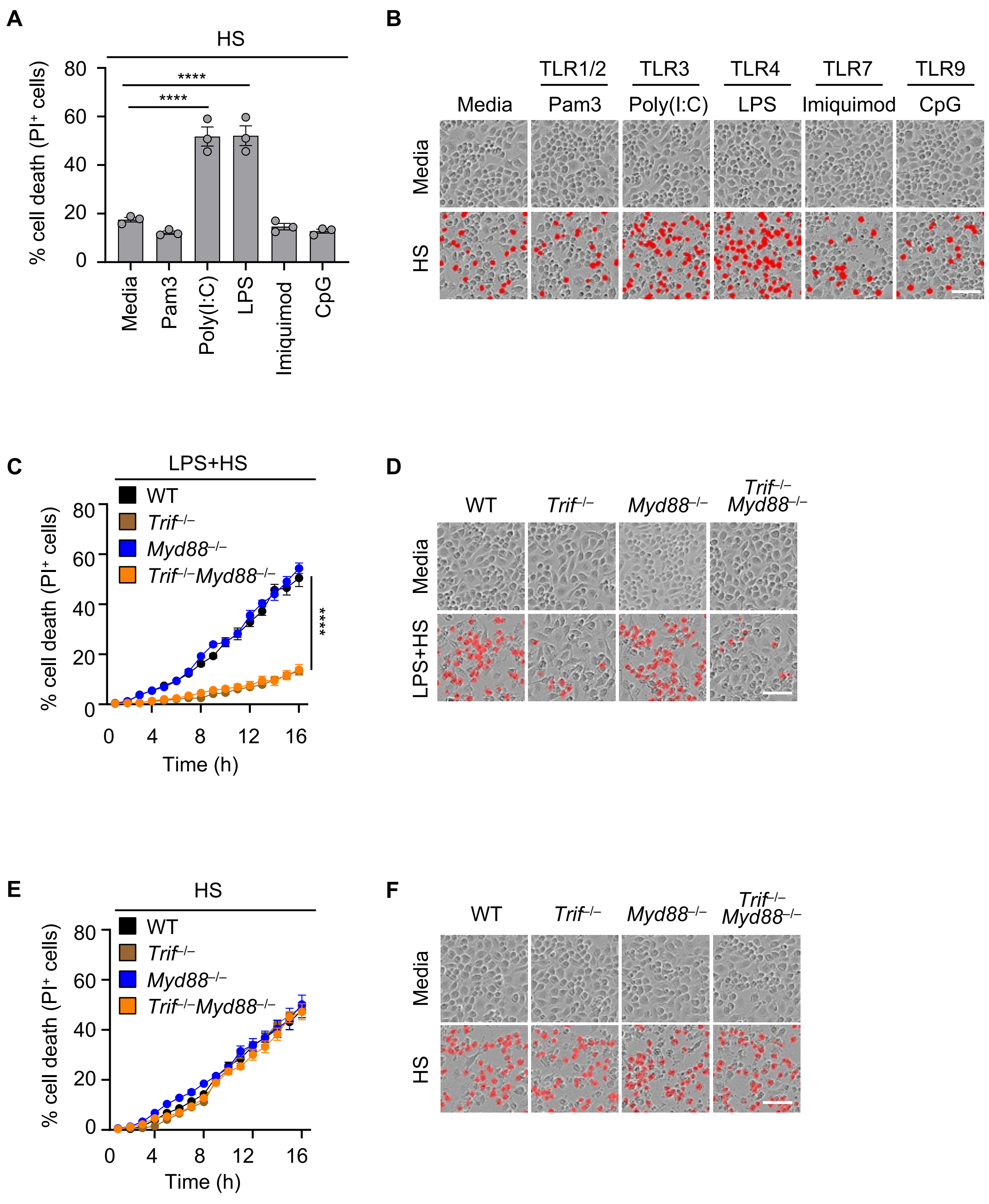

Supplementary Fig. 2. TLR and TRIF signaling promote PAMP plus heat stress-induced cell death

A Analysis of cell death in Pam3CSK4 (Pam3), poly(I:C), lipopolysaccharide (LPS), imiquimod, or CpG-primed wild type (WT) bone marrow-derived macrophages (BMDMs) at 16 h post-treatment (hpt) with heat stress (HS; 43 °C for 30 min). **B** Representative images of cell death in

(A) at 16 hpt. **C** Real-time analysis of cell death in LPS-primed WT, *Trif*$^{-/-}$, *Myd88*$^{-/-}$, and *Trif*$^{-/-}$*Myd88*$^{-/-}$ BMDMs following challenge with HS (43 °C for 30 min). **D** Representative images of cell death in (C) at 16 hpt. **E** Real-time analysis of cell death in WT, *Trif*$^{-/-}$, *Myd88*$^{-/-}$, and *Trif*$^{-/-}$*Myd88*$^{-/-}$ BMDMs following HS (43 °C for 60 min). **F** Representative images of cell death in (E) at 16 hpt. **A, C, E** Data are shown as mean ± SEM; ****P < 0.0001 (one-way ANOVA with Bonferroni's multiple comparisons test; **A** n = 3 from 3 biologically independent samples and **C, E** n = 4 from 4 biologically independent samples). **B, D, F** Images are representative of at least three independent experiments. Scale bar, 50 µm. Exact *P* values are presented in Supplementary Data file 2.

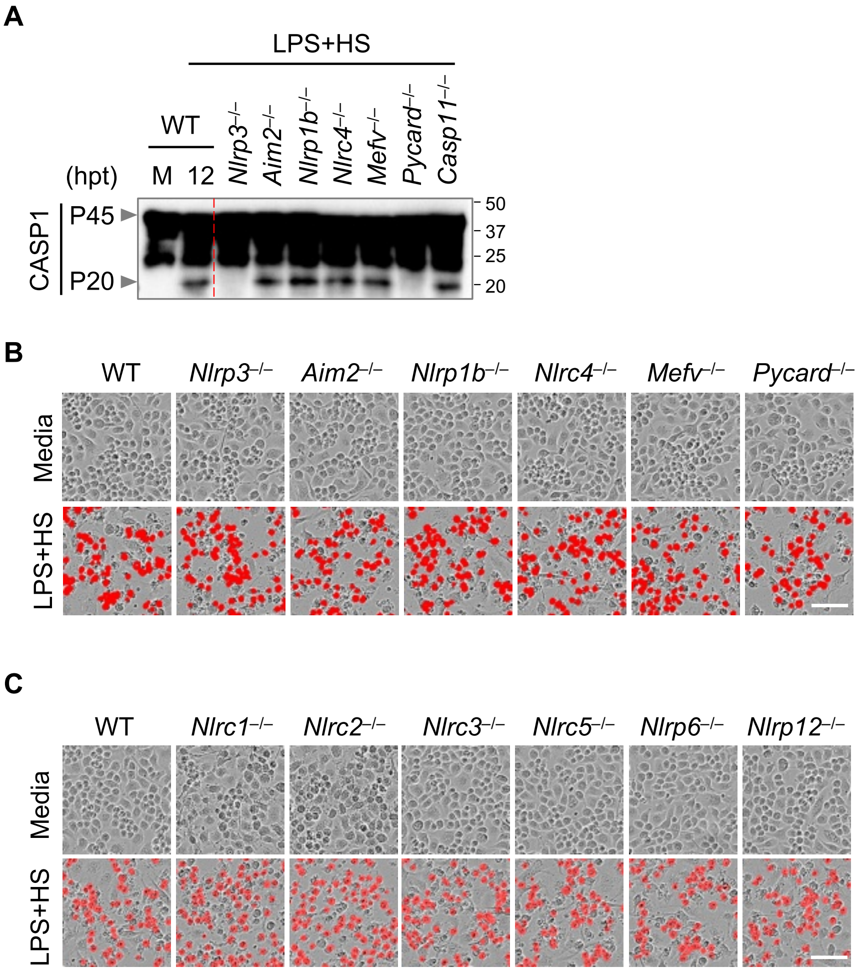

Supplementary Fig. 3. LPS plus heat stress activates the NLRP3 inflammasome

A Immunoblot analysis of pro- (P45) and activated (P20) caspase-1 (CASP1) in lipopolysaccharide (LPS)-primed bone marrow-derived macrophages (BMDMs) obtained from the indicated genotypes at 12 h post-treatment (hpt) with heat stress (HS; 43 °C, 30 min). M, media control. **B and C** Representative images of cell death in LPS-primed BMDMs obtained from the indicated genotypes at 16 hpt (43 °C, 30 min). Scale bar, 50 μm (B and C). **A–C** Images are representative of at least three independent experiments. For uncropped western blots, see the accompanying source data.

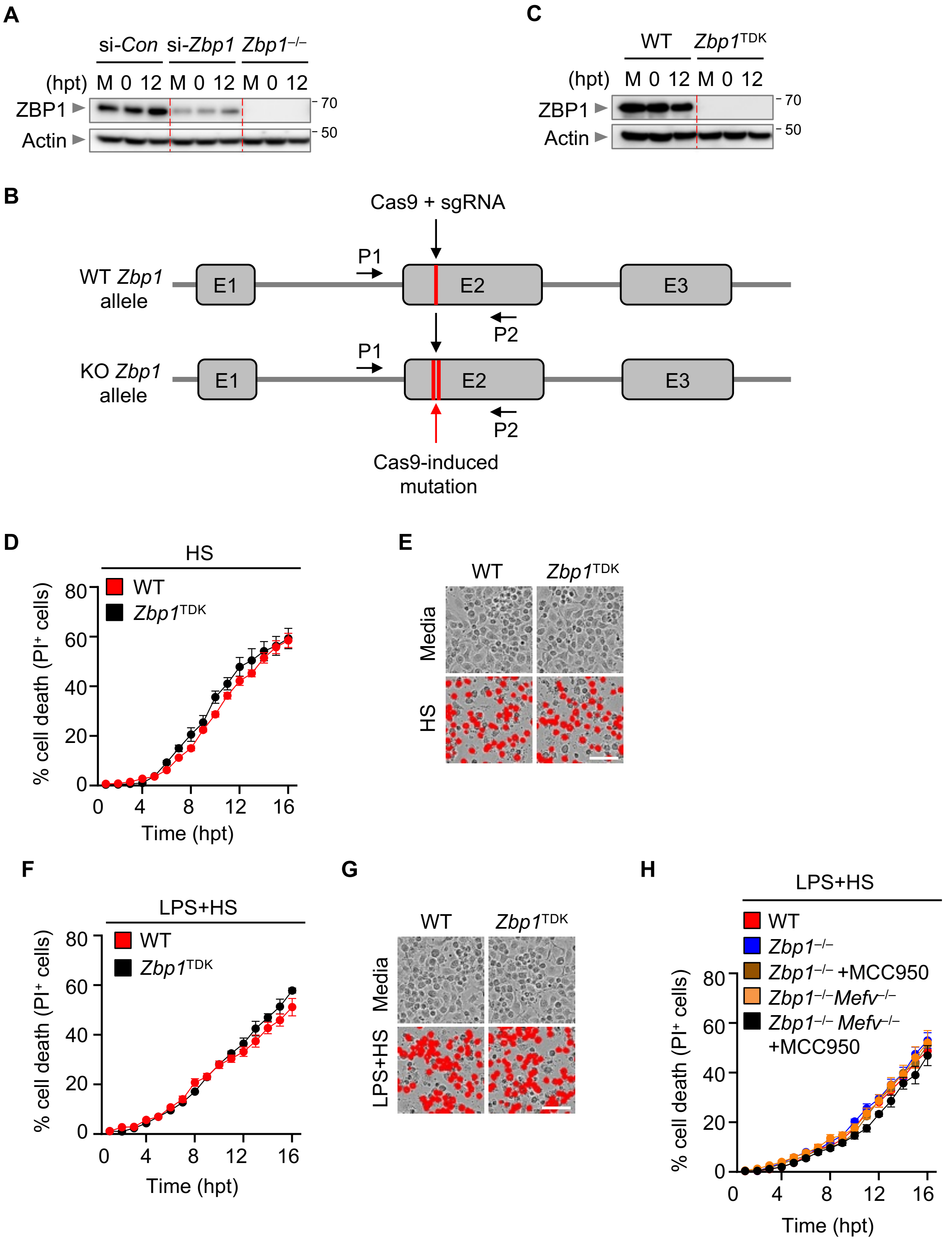

Supplementary Fig. 4. ZBP1 is not required for cell death in response to heat stress or LPS plus heat stress

A Immunoblot analysis of ZBP1 in lipopolysaccharide (LPS)-primed wild type (WT) bone marrow-derived macrophages (BMDMs) treated with control siRNA (si-*Con*) or *Zbp1* siRNA (si-*Zbp1*) and *Zbp1*$^{-/-}$ BMDMs at the indicated timepoints after heat stress (HS; 43 °C, 30 min). M, media control.

Actin is used as the internal control. **B** The strategy for generation of the *Zbp1*^{TDK} mice is shown. One sgRNA targeting exon 2 was used to disrupt the *Zbp1* locus as described in the materials and methods section (depictions are not to scale). The vertical bar in red denotes the sgRNA target location in the genomic sequence. The location of the genomic mutation is highlighted by a red vertical double-bar in the knockout (KO) *Zbp1* allele, and the primer binding locations for Sanger sequencing in the WT and KO alleles are depicted using short black arrows. **C** Immunoblot analysis of ZBP1 in LPS-primed WT and *Zbp1*^{TDK} BMDMs at the indicated timepoints after HS (43 °C, 30 min). Actin is used as the internal control. **D** Real-time analysis of cell death in WT and *Zbp1*^{TDK} BMDMs following HS (43 °C, 60 min). **E** Representative images of cell death in (D) at 16 h post-treatment (hpt). **F** Real-time analysis of cell death in LPS-primed WT and *Zbp1*^{TDK} BMDMs following HS (43 °C, 30 min). **G** Representative images of cell death in (F) at 16 hpt. **H** Real-time analysis of cell death in LPS-primed WT, *Zbp1*$^{-/-}$, and *Zbp1*$^{-/-}$*Mefv*$^{-/-}$ BMDMs without or with MCC950 following HS (43 °C, 30 min). **A, C, E, G** Images are representative of at least three independent experiments. **E, G** Scale bar, 50 µm. **D, F, H** Data are shown as mean ± SEM (one-way ANOVA with Bonferroni's multiple comparisons test; n = 4 from 4 biologically independent samples). Exact *P* values are presented in Supplementary Data file 2. For uncropped western blots, see the accompanying source data.

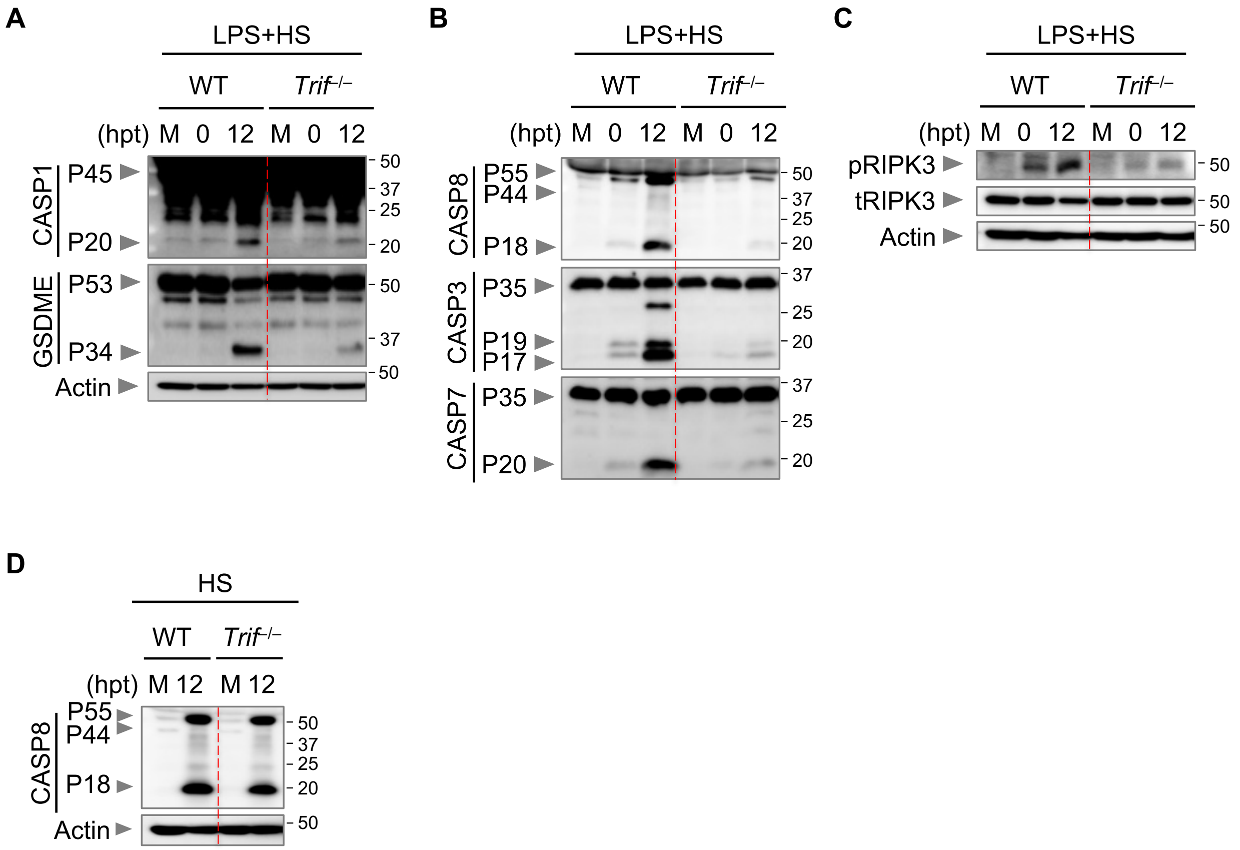

Supplementary Fig. 5. LPS plus heat stress-induced activation of PANoptosis molecules is TRIF-dependent

A–C Immunoblot analysis of (A) pro- (P45) and activated (P20) caspase-1 (CASP1); pro- (P53) and activated (P34) gasdermin E (GSDME); (B) pro- (P55) and cleaved (P44 and P18) caspase-8 (CASP8); pro- (P35) and cleaved (P19 and P17) caspase-3 (CASP3); pro- (P35) and cleaved (P20) caspase-7 (CASP7); (C) phosphorylated receptor-interacting serine/threonine kinase 3 (pRIPK3), and total RIPK3 (tRIPK3) in LPS-primed wild type (WT) and *Trif*$^{-/-}$ bone marrow-derived macrophages (BMDMs) at the indicated timepoints after HS (43 °C for 30 min). **D** Immunoblot analysis of pro- (P55) and cleaved (P44 and P18) CASP8 in WT and *Trif*$^{-/-}$ BMDMs at the indicated timepoints after HS (43 °C for 60 min). M, media control. Actin is used as the internal control. **A–D** Images are representative of at least three independent experiments. For uncropped western blots, see the accompanying source data.

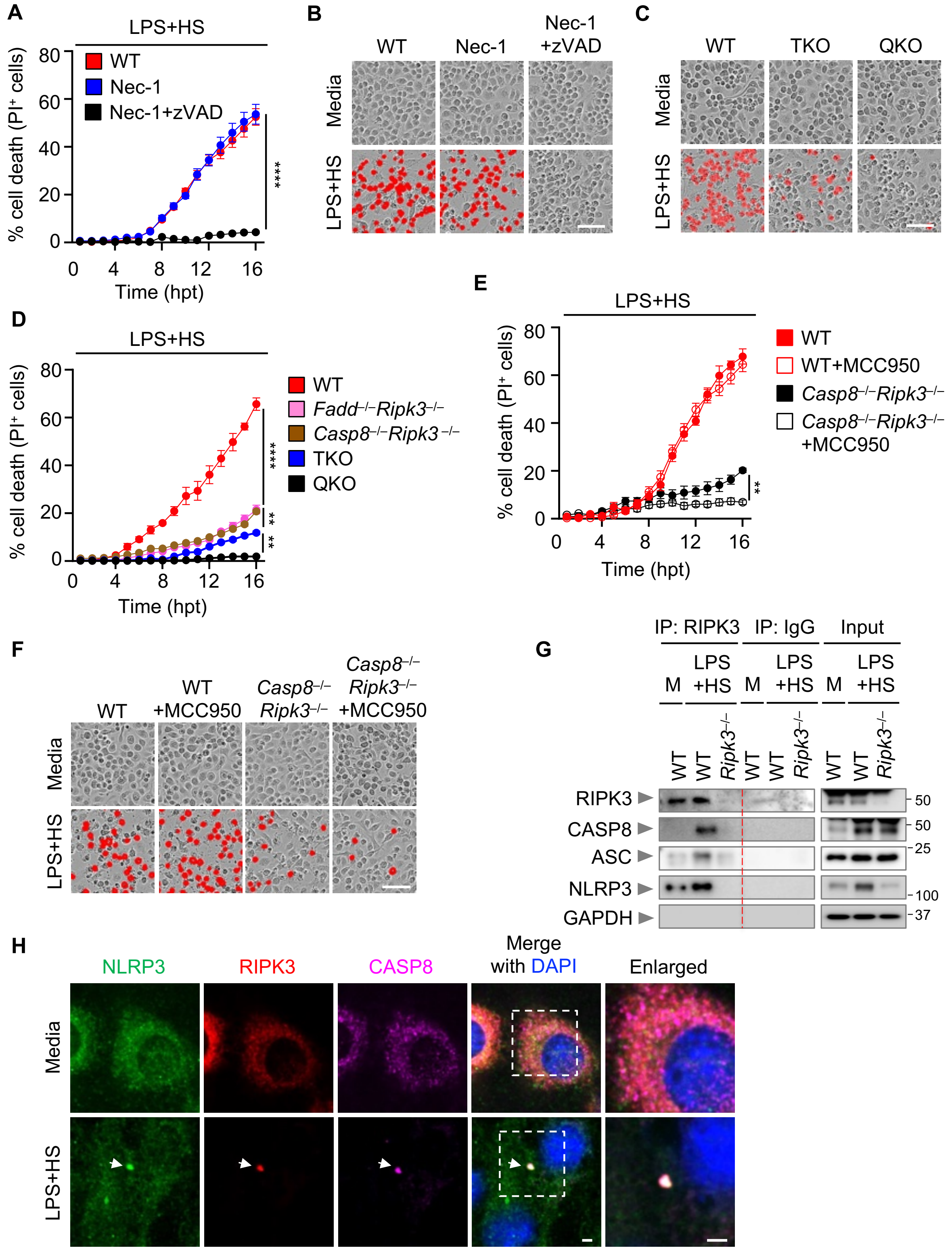

Supplementary Fig. 6. Inhibition of caspases and RIPKs protects against LPS plus heat stress-induced PANoptosis

A Real-time analysis of cell death in lipopolysaccharide (LPS)-primed wild type (WT) bone marrow-derived macrophages (BMDMs) with or without Nec-1 and/or z-VAD-FMK (zVAD)

treatment following heat stress (HS; 43 °C, 30 min). **B** Representative images of cell death in (A) at 16 h post-treatment (hpt). **C** Representative images of cell death in LPS-primed WT, *Casp1*$^{-/-}$*Casp8*$^{-/-}$*Ripk3*$^{-/-}$ (TKO), and *Casp1*$^{-/-}$*Casp11*$^{-/-}$*Casp8*$^{-/-}$*Ripk3*$^{-/-}$ (QKO) BMDMs challenged with HS (43 °C, 30 min). **D** Real-time analysis of cell death in LPS-primed WT, *Fadd*$^{-/-}$*Ripk3*$^{-/-}$, *Casp8*$^{-/-}$*Ripk3*$^{-/-}$, TKO, and QKO BMDMs challenged with HS (43 °C, 30 min). **E** Real-time analysis of cell death in LPS-primed WT and *Casp8*$^{-/-}$*Ripk3*$^{-/-}$ BMDMs without or with MCC950 following HS (43 °C, 30 min). **F** Representative images of cell death in (E) at 16 hpt. **G** Immunoprecipitation (IP) in LPS-primed WT and *Ripk3*$^{-/-}$ BMDMs 12 h after HS (43 °C, 30 min) with anti-RIPK3 or IgG control antibodies. Immunoblot analysis of RIPK3, caspase-8 (CASP8), ASC, NLRP3, and GAPDH. M, media control. GAPDH is used as the internal control. **H** Immunofluorescence images of WT LPS-primed BMDMs at 12 h post-treatment (hpt) (43 °C for 30 min). Nuclei were stained with DAPI. Arrowheads indicate the colocalized NLRP3, RIPK3, and CASP8 specks. **A, D, E** Data are shown as mean ± SEM; $^{**}P < 0.01$ and $^{****}P < 0.0001$ (one-way ANOVA with Bonferroni's multiple comparisons test; n = 4 from 4 biologically independent samples). **B, C, F, G, H** Images are representative of at least three independent experiments. **B, C, F** Scale bar, 50 μm. **H** Scale bar, 5 μm. Exact *P* values are presented in Supplementary Data file 2. For uncropped western blots, see the accompanying source data.

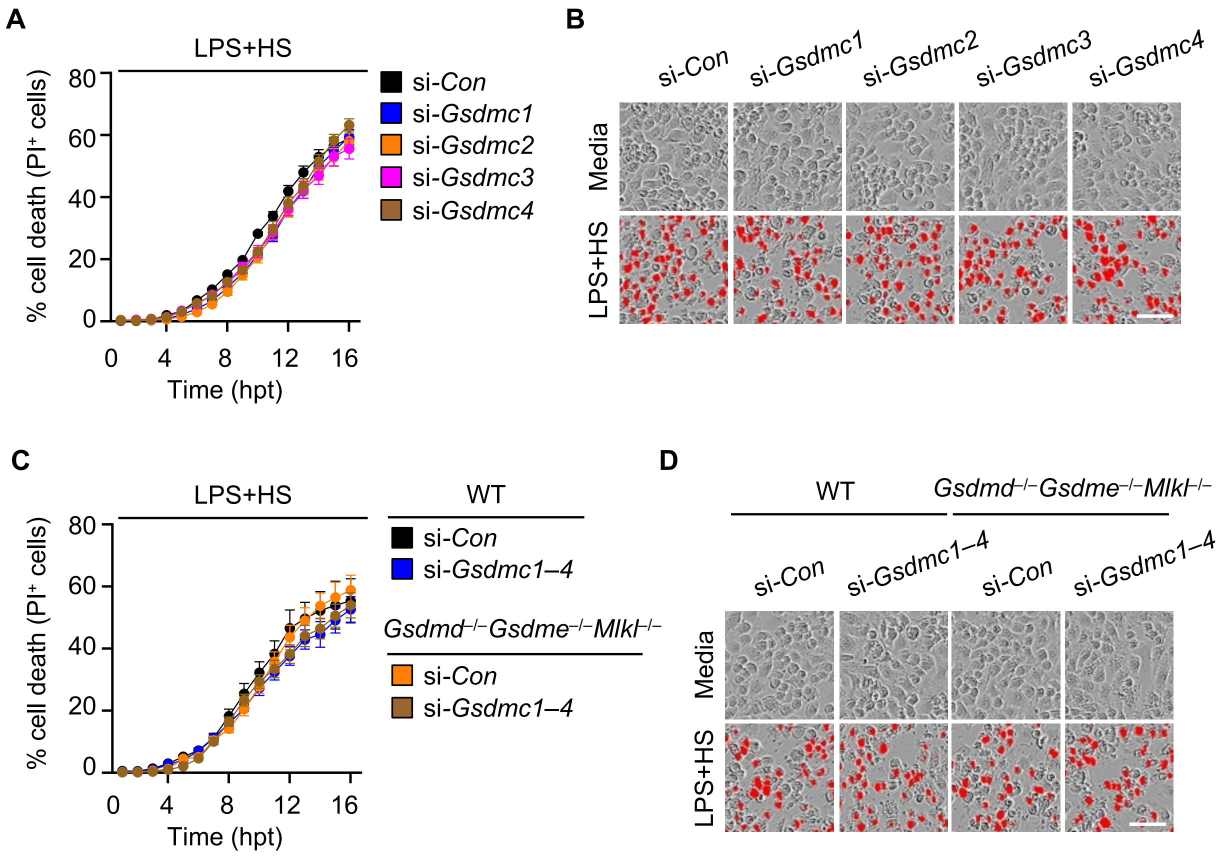

Supplementary Fig. 7. GSDMC does not contribute to LPS plus heat stress-induced PANoptosis

A Real-time analysis of cell death in wild type (WT) bone marrow-derived macrophages (BMDMs) treated with control siRNA (si-*Con*), *Gsdmc1* siRNA (si-*Gsdmc1*), *Gsdmc2* siRNA (si-*Gsdmc2*), *Gsdmc3* siRNA (si-*Gsdmc3*), or *Gsdmc4* siRNA (si-*Gsdmc4*) followed by lipopolysaccharide (LPS) priming and heat stress (HS; 43 °C, 30 min) challenge. **B** Representative images of cell death in (A) at 16 h post-treatment (hpt). **C** Real-time analysis of cell death in WT and *Gsdmd*$^{-/-}$ *Gsdme*$^{-/-}$*Mlkl*$^{-/-}$ BMDMs treated with si-*Con* or *Gsdmc1–4* siRNA (si-*Gsdmc1–4*) followed by LPS priming and HS (43 °C, 30 min) challenge. **D** Representative images of cell death in (C) at 16 hpt. **A, C** Data are shown as mean ± SEM (one-way ANOVA with Bonferroni's multiple comparisons test; n = 4 from 4 biologically independent samples). **B, D** Images are representative of at least three independent experiments. Scale bar, 50 μm. Exact *P* values are presented in Supplementary Data file 2.

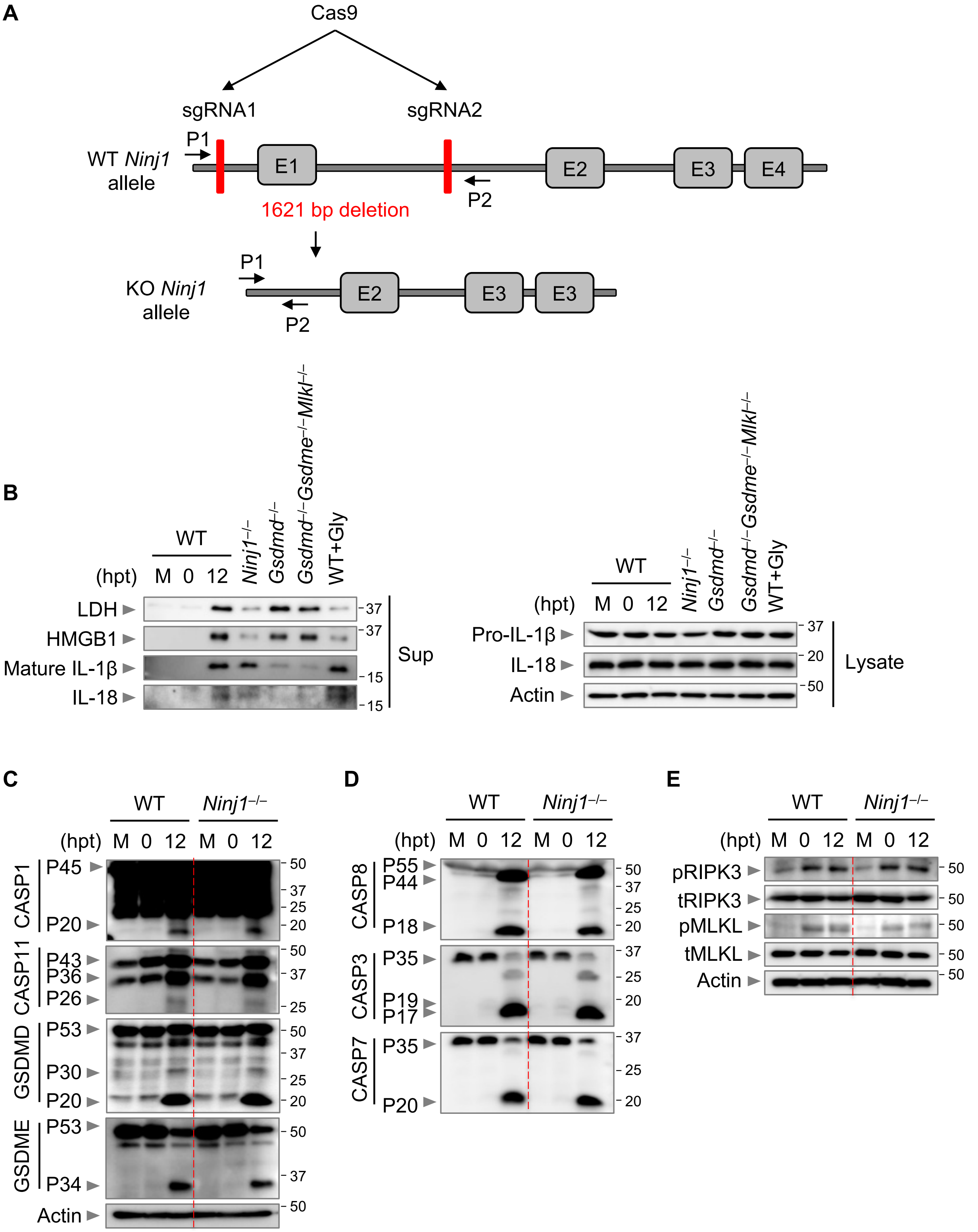

Supplementary Fig. 8. NINJ1 is not required for activation of PANoptosis molecules

A The strategy for the generation of *Ninj1*$^{-/-}$ mice is shown. Two sgRNAs targeting the *Ninj1* gene as depicted in the figure were used to disrupt the *Ninj1 locus* as described in the materials and

methods section (depictions are not to scale). The red vertical bars denote the sgRNA targeting locations in the genomic sequence. The genotyping primer binding locations in the wild type (WT) and knockout (KO) alleles are depicted using short black arrows. **B** Immunoblot analysis of LDH, HMGB1, IL-1β, and IL-18 in supernatant (Sup) or lysate from lipopolysaccharide (LPS)-primed WT, *Ninj1*$^{-/-}$, *Gsdmd*$^{-/-}$, *Gsdmd*$^{-/-}$*Gsdme*$^{-/-}$*Mlkl*$^{-/-}$, or glycine (Gly)-treated WT bone marrow-derived macrophages (BMDMs) at 12 h or the indicated timepoints after heat stress (HS; 43 °C, 30 min). M, media control. **C–E** Immunoblot analysis of (C) pro- (P45) and activated (P20) caspase-1 (CASP1); pro- (P43) and cleaved (P36 and P26) caspase-11 (CASP11); pro- (P53), activated (P30), and inactivated (P20) gasdermin D (GSDMD); pro- (P53) and activated (P34) gasdermin E (GSDME); (D) pro- (P55) and cleaved (P44 and P18) caspase-8 (CASP8); pro- (P35) and cleaved (P19 and P17) caspase-3 (CASP3); pro- (P35) and cleaved (P20) caspase-7 (CASP7); and (E) phosphorylated receptor-interacting serine/threonine kinase 3 (pRIPK3), total RIPK3 (tRIPK3), phosphorylated mixed lineage kinase domain-like (pMLKL), and total MLKL (tMLKL) in LPS-primed WT and *Ninj1*$^{-/-}$ BMDMs at the indicated time points after HS (43 °C for 30 min). M, media control. Actin is used as the internal control. **B–E** Images are representative of at least three independent experiments. For uncropped western blots, see the accompanying source data.

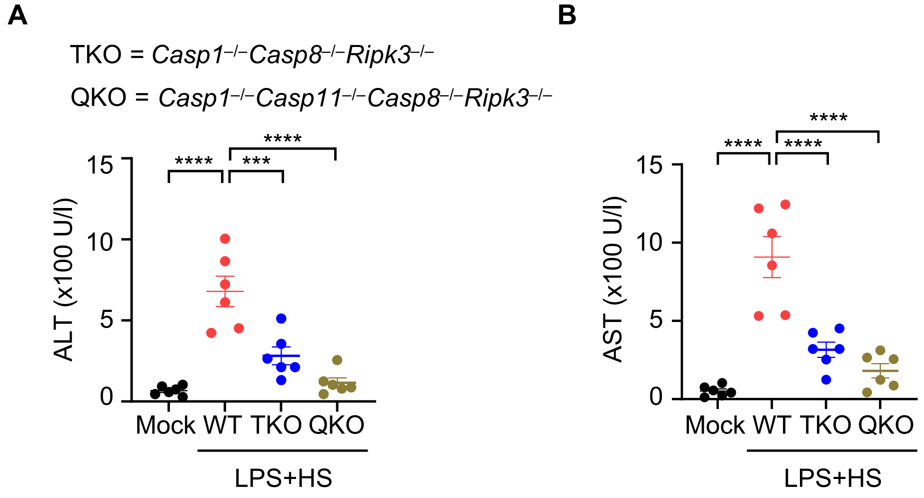

Supplementary Fig. 9. In vivo heat stress model induces pathology that is rescued by blocking inflammatory cell death, PANoptosis

A,B Analysis of (A) ALT and (B) AST levels in the serum of untreated wild type (WT) mice (mock, n = 6) or WT (n = 6), *Casp1*$^{-/-}$*Casp8*$^{-/-}$*Ripk3*$^{-/-}$ (TKO; n = 6), and *Casp1*$^{-/-}$*Casp11*$^{-/-}$*Casp8*$^{-/-}$*Ripk3*$^{-/-}$ (QKO; n = 6) mice injected with lipopolysaccharide (LPS) (5 mg/kg body weight) for 2 h, followed by heat stress (HS) at 39 °C for 2 h. The serum was collected at 36 h post-HS. Data are pooled from three independent experiments. Data are shown as mean ± SEM; ****P* < 0.001, and *****P* < 0.0001 (one-way ANOVA with Bonferroni's multiple comparisons test). Exact *P* values are presented in Supplementary Data file 2.
