# Supplementary material for: NINJ1 mediates inflammatory cell death, PANoptosis, and lethality during infection conditions and heat stress
Source: Nat Commun. 2024 Feb 26;15:1739. doi: 10.1038/s41467-024-45466-x (PMC10897308; doi:10.1038/s41467-024-45466-x)
Supplement: Supplementary file 3 — Description of Additional Supplementary Files [file 41467_2024_45466_MOESM3_ESM.pdf]

Description of Additional Supplementary Files

Supplementary Data 1: Results from the CRISPR screen for cell death molecules

Supplementary Data 2: Exact P values for all figures
