# Supplementary material for: NINJ1 mediates inflammatory cell death, PANoptosis, and lethality during infection conditions and heat stress
Source: Nat Commun. 2024 Feb 26;15:1739. doi: 10.1038/s41467-024-45466-x (PMC10897308; doi:10.1038/s41467-024-45466-x)
Supplement: Supplementary file 6 — Reporting Summary [file 41467_2024_45466_MOESM6_ESM.pdf]

nature portfolio

Corresponding author(s): Thirumala-Devi Kanneganti

Last updated by author(s): Feb 3, 2024

Reporting Summary

Nature Portfolio wishes to improve the reproducibility of the work that we publish. This form provides structure for consistency and transparency in reporting. For further information on Nature Portfolio policies, see our Editorial Policies and the Editorial Policy Checklist.

Statistics

For all statistical analyses, confirm that the following items are present in the figure legend, table legend, main text, or Methods section.

n/a	Confirmed	
☐	☒	The exact sample size (*n*) for each experimental group/condition, given as a discrete number and unit of measurement
☐	☒	A statement on whether measurements were taken from distinct samples or whether the same sample was measured repeatedly
☐	☒	The statistical test(s) used AND whether they are one- or two-sided<br>*Only common tests should be described solely by name; describe more complex techniques in the Methods section.*
☒	☐	A description of all covariates tested
☐	☒	A description of any assumptions or corrections, such as tests of normality and adjustment for multiple comparisons
☐	☒	A full description of the statistical parameters including central tendency (e.g. means) or other basic estimates (e.g. regression coefficient) AND variation (e.g. standard deviation) or associated estimates of uncertainty (e.g. confidence intervals)
☐	☒	For null hypothesis testing, the test statistic (e.g. *F*, *t*, *r*) with confidence intervals, effect sizes, degrees of freedom and *P* value noted<br>*Give P values as exact values whenever suitable.*
☒	☐	For Bayesian analysis, information on the choice of priors and Markov chain Monte Carlo settings
☒	☐	For hierarchical and complex designs, identification of the appropriate level for tests and full reporting of outcomes
☒	☐	Estimates of effect sizes (e.g. Cohen's *d*, Pearson's *r*), indicating how they were calculated

Our web collection on statistics for biologists contains articles on many of the points above.

Software and code

Policy information about availability of computer code

Data collection	1. The kinetics of cell death and microscopy images were collected with the IncuCyte live-cell imaging automated system using the IncuCyte S3 (Sartorius, v2020) software.<br>2. Confocal microscopy samples were visualized and imaged using the Marianas spinning disc confocal system (Intelligent Imaging Innovations) comprised of an inverted AxioObserver Z.1 microscope (Carl Zeiss), CSU-W1 with SoRa (Yokogawa), Prime95B sCMOS camera (Photometrics), 405 nm, 473 nm, 561 nm, 647 nm solid state laser lines (Coherent), a 1.4NA 100 × oil objective, and Slidebook 6 software.<br>3. For immunoblotting, membranes were developed with an Amersham imager.<br>4. For NGS sequencing, single end 100 cycle sequencing was performed on a NovaSeq 6000 (Illumina).
Data analysis	1. For immunoblotting, images were analyzed with ImageJ (v1.53a).<br>2. The kinetics of cell death was analyzed using the IncuCyte S3 (Sartorius, v2020) software.<br>3. Graphpad Prism version 9 was used for data analysis.<br>4. Validation to check gRNA presence and representation was performed using calc_auc_v1.1.py.<br>5. CRISPR KO screens were analyzed using Mageck-Vispr v0.5.7.<br>6. The top gene hits along with their significance from the CRISPR screen were highlighted using a volcano plot using MAGeCKFlute v2.0.0.<br>7. Quality control steps of GEO dataset were performed, including normalised quantiles using the 'normalize.quantiles' function from preprocessCore v1.58.0 package when the counts are not normalized, followed by log2 transformation for downstream differential expression analysis.<br>8. Differential expression analysis was performed using the limma v3.52.1 package in R v4.1.1.

For manuscripts utilizing custom algorithms or software that are central to the research but not yet described in published literature, software must be made available to editors and reviewers. We strongly encourage code deposition in a community repository (e.g. GitHub). See the Nature Portfolio guidelines for submitting code & software for further information.

Data

Policy information about availability of data

All manuscripts must include a data availability statement. This statement should provide the following information, where applicable:

- Accession codes, unique identifiers, or web links for publicly available datasets
- A description of any restrictions on data availability
- For clinical datasets or third party data, please ensure that the statement adheres to our policy

Next-generation sequencing results from the CRISPR screen are deposited in the Gene Expression Omnibus (GEO) database under accession code GSE252609 (https://www.ncbi.nlm.nih.gov/geo/query/acc.cgi?acc=GSE252609). All other datasets generated and analyzed in this study are provided within the manuscript and the accompanying supplementary figures, tables, and data files. Source data are provided with this paper. The publicly available dataset from cancer cells subjected to hyperthermia was re-analyzed for this study and is available in the GEO database under accession code GSE48398 (https://www.ncbi.nlm.nih.gov/geo/query/acc.cgi?acc=GSE48398), originally published by Amaya, C et al. (ref. 40).

Research involving human participants, their data, or biological material

Policy information about studies with human participants or human data. See also policy information about sex, gender (identity/presentation), and sexual orientation and race, ethnicity and racism.

Reporting on sex and gender	Fresh human blood was collected from the apheresis rings of anonymous healthy blood donors from the blood bank at St. Jude Children's Research Hospital. Patient sex and gender remain blinded to the researchers.
Reporting on race, ethnicity, or other socially relevant groupings	Due to the anonymity of the blood donors, no population characteristics were obtained.
Population characteristics	Healthy blood donors volunteer to provide blood at the St. Jude Children's Research Hospital blood bank.
Recruitment	Healthy blood donors volunteer to provide blood at the St. Jude Children's Research Hospital blood bank.
Ethics oversight	Blood collection and apheresis ring collection were conducted under protocols approved by the St. Jude IRB.

Note that full information on the approval of the study protocol must also be provided in the manuscript.

Field-specific reporting

Please select the one below that is the best fit for your research. If you are not sure, read the appropriate sections before making your selection.

☒ Life sciences ☐ Behavioural & social sciences ☐ Ecological, evolutionary & environmental sciences

For a reference copy of the document with all sections, see nature.com/documents/nr-reporting-summary-flat.pdf

Life sciences study design

All studies must disclose on these points even when the disclosure is negative.

Sample size	No statistical method was used to determine sample size. The sample size differed among experiments and it is reported throughout the manuscript and in the legend of each figure. If all 3 replicates gave similar results, experiments were considered as reproducible and completed.
Data exclusions	ROUT test with Q-value of 0.1 as a cut-off was used to exclude outliers. The data exclusion criterion was pre-established. All data were retained if removing outliers could have resulted in a sample size of less than 3. No outliers were removed from the datasets reported.
Replication	Each experiment was performed with at least 3 biological replicates. All the reported results are from experiments in which every repeat gave similar results.
Randomization	For in vitro experiments, cells from the same pool of BMDMs were randomly split into separate wells and subjected to the treatments. For in vivo experiments, animals from the same cage were randomly selected by matching sex and age for treatment.
Blinding	Investigators were not blinded. None of the reported experiments require subjective decision making. Key experiments were repeated by independent researchers. Therefore, there was no need for blinding.

Reporting for specific materials, systems and methods

We require information from authors about some types of materials, experimental systems and methods used in many studies. Here, indicate whether each material, system or method listed is relevant to your study. If you are not sure if a list item applies to your research, read the appropriate section before selecting a response.

Materials & experimental systems

n/a	Involved in the study	
	☒	Antibodies
	☒	Eukaryotic cell lines
☒		Palaeontology and archaeology
	☒	Animals and other organisms
☒		Clinical data
☒		Dual use research of concern
☒		Plants

Methods

n/a	Involved in the study	
☒		ChIP-seq
☒		Flow cytometry
☒		MRI-based neuroimaging

Antibodies

Antibodies used

Immunoblotting: anti-caspase-1 (AdipoGen, AG-20B-0044, 1:1000), anti-caspase-11 (Novus Biologicals, NB120-10454, 1:1,000), anti-caspase-3 (CST, #9662, 1:1000), anti-cleaved caspase-3 (CST, #9661, 1:1000), anti-caspase-7 (CST, #9492, 1:1000), anti-cleaved caspase-7 (CST, #9491, 1:1000), anti-caspase-8 (CST, #4927, 1:1000), anti-cleaved caspase-8 (CST, #8592, 1:1000), anti-pRIPK3 (CST, #91702S, 1:1000), anti-RIPK3 (ProSci, #2283, 1:1000), anti-pMLKL (CST, #37333, 1:1000), anti-MLKL (Abgent, AP14272b, 1:1000), anti-GSDMD (Abcam, ab209845, 1:1000), anti-GSDME (Abcam, ab215191, 1:1000), anti-NLRP3 (Adipogen, AG-20B-0014-C100, 1:1000), anti-ZBP1 (AdipoGen, AG-20B-0010, 1:1000), anti-NINJ1 (Customized, Ref. 83, 1:1,000), anti-LDHA (Proteintech, 19987-1-AP, 1:1,000), anti-HMGB1 (Abcam, ab79823, 1:1,000), anti-cleaved IL-1β (CST, 63124, 1:1,000), anti-IL-1β (CST 12426, 1:1,000), anti-IL-18 (Abcam, ab207323, 1:1,000), anti-ASC (Adipogen, AG-25B-0006, 1:1,000), anti-GAPDH (Santacruz, sc-166574 HRP, 1:10,000), anti-β-actin (Proteintech, 66009-1-IG, 1:5,000); anti-IgG (CST, #3900); secondary anti-rabbit (111-035-047), anti-mouse (315-035-047) or anti-rat (112-035-003) HRP antibodies from Jackson ImmunoResearch Laboratories.

Microscopy: anti-ASC (Millipore, 04-147, 1:100), anti-RIPK3 (Millipore, MABC1595, 1:200), anti-CASP8 (CST, #4927, 1:200), anti-NLRP3 (Adipogen, AG-20B-0014-C100, 1:200) or anti-NINJ1 (Customized, 1:200), Alexa Fluor 488 (ThermoFisher, A20181)-conjugated anti-ASC (Millipore, 04-147), Alexa Fluor 488-conjugated antibody against mouse immunoglobulin G (IgG) (Thermo Fisher Scientific, A11029, 1:200), Alexa Fluor 568-conjugated antibody against rat IgG (Thermo Fisher Scientific, A11077, 1:200), or Alexa Fluor 647-conjugated antibody against rabbit IgG (Thermo Fisher Scientific, A21245, 1:200)

Validation

Antibodies were validated by their source company.

1. anti-caspase-1 (AdipoGen, AG-20B-0044): Species: Mouse, Rat; Application: WB, IHC, IP; Citation: Measuring the inflammasome: O. Gross; Methods Mol. Biol. (2012).; Website: https://adipogen.com/ag-20b-0042-anti-caspase-1-p20-mouse-mab-casper-1.html
2. anti-caspase-11 (Novus Biologicals, NB120-10454): Species: Human, Mouse, Rat; Application: WB, ELISA, ICC, IHC, IP; Citation: Endocytic membrane repair by ESCRT-III controls antigen export to the cytosol during antigen cross-presentation: G. Marine; Cell Rep. (2022).; Website: https://www.novusbio.com/products/caspase-11-antibody-17d9_nb120-10454#reviews-publications
3. anti-caspase-3 (CST, #9662): Species: Human, Mouse, Rat; Application: WB, IHC, IP; Citation: Type I interferon signaling mediates Mycobacterium tuberculosis-induced macrophage death: L. Zhang; J. Exp. Med. (2021).; Website: https://www.cellsignal.com/products/primary-antibodies/caspase-3-antibody/9662
4. anti-cleaved caspase-3 (CST, #9661): Species: Human, Mouse, Rat; Application: WB, IHC, IP, IF; Citation: RIPK1 Distinctly Regulates Yersinia-Induced Inflammatory Cell Death, PANoptosis: R K Subbarao Malireddi; Immunohorizons (2020).; Website: https://www.cellsignal.com/products/primary-antibodies/cleaved-caspase-3-asp175-antibody/9661
5. anti-caspase-7 (CST, #9492): Species: Human, Mouse, Rat; Application: WB; Citation: RIPK1 Distinctly Regulates Yersinia-Induced Inflammatory Cell Death, PANoptosis: R K Subbarao Malireddi; Immunohorizons (2020).; Website: https://www.cellsignal.com/products/primary-antibodies/caspase-7-antibody/9492
6. anti-cleaved caspase-7 (CST, #9491): Species: Human, Mouse, Rat; Application: WB, IP; Citation: RIPK1 Distinctly Regulates Yersinia-Induced Inflammatory Cell Death, PANoptosis: R K Subbarao Malireddi; Immunohorizons (2020).; Website: https://www.cellsignal.com/products/primary-antibodies/cleaved-caspase-7-asp198-antibody/9491
7. anti-caspase-8 (CST, #4927): Species: Mouse; Application: WB; Citation: Caspase-6 Is a Key Regulator of Innate Immunity, Inflammasome Activation, and Host Defense:
Min Zheng; Cell (2020).; Website: https://www.cellsignal.com/products/primary-antibodies/caspase-8-antibody-mouse-specific/4927
8. anti-cleaved caspase-8 (CST, #8592): Species: Mouse; Application: WB; Citation: Caspase-6 Is a Key Regulator of Innate Immunity, Inflammasome Activation, and Host Defense: Min Zheng; Cell (2020).; Website: https://www.cellsignal.com/products/primary-antibodies/caspase-8-antibody-mouse-specific/8592
9. anti-pRIPK3 (CST, #91702S): Species: Mouse; Application: WB, IF; Citation: Diverse sequence determinants control human and mouse receptor interacting protein 3 (RIP3) and mixed lineage kinase domain-like (MLKL) interaction in necroptotic signaling: Wanze Chen; J. Biol. Chem. (2013).; Website: https://www.cellsignal.com/products/primary-antibodies/phospho-rip3-thr231-ser232-e7s1r-rabbit-mab/91702?site-search-type=Products&N=4294956287&Ntt=91702s&fromPage=plp&_requestid=4790034
10. anti-RIPK3 (ProSci, #2283): Species: Human, Mouse, Rat; Application: WB, IHC, IP, IF; Citation: Receptor interacting protein kinase-3 determines cellular necrotic response to TNF-alpha: Sudan He; Cell (2009).; Website: https://www.prosci-inc.com/rip3-antibody-2283.html
11. anti-pMLKL (CST, #37333): Species: Mouse; Application: WB, IF; Citation: Caspase-6 Is a Key Regulator of Innate Immunity, Inflammasome Activation, and Host Defense. Min Zheng; Cell (2020).; Website: https://www.cellsignal.com/products/primary-antibodies/phospho-mlkl-ser345-d6e3g-rabbit-mab/37333
12. anti-MLKL (Abgent, AP14272b): Species: Mouse; Application: WB; Citation: Caspase-6 Is a Key Regulator of Innate Immunity, Inflammasome Activation, and Host Defense. Min Zheng; Cell (2020).; Website: https://www.citeab.com/antibodies/240195-ap14272b-m-mlkl-antibody-c-term
13. anti-GSDMD (Abcam, ab209845): Species: Mouse; Application: WB, IP; Citation: Caspase-6 Is a Key Regulator of Innate Immunity, Inflammasome Activation, and Host Defense: Min Zheng; Cell (2020).; Website: https://www.abcam.com/gsdmd-antibody-epr19828-

ab209845.html
14. anti-GSDME (Abcam, ab215191): Species: Human, Mouse, Rat; Application: WB, IP; Citation: Structural Mechanism for GSDMD Targeting by Autoprocessed Caspases in Pyroptosis: Kun Wang; Cell (2020).; Website: https://www.abcam.com/dfna5gsdme-antibody-epr19859-n-terminal-ab215191.html
15. anti-ZBP1 (AdipoGen, AG-20B-0010): Species: Human, Mouse; Application: WB, IP, ICC; Citation: Caspase-6 Is a Key Regulator of Innate Immunity, Inflammasome Activation, and Host Defense: Min Zheng; Cell (2020).; Website: https://adipogen.com/ag-20b-0010-anti-zbp1-mab-zippy-1.html
16. anti-ASC (Adipogen, AG-25B-0006, 1:1000): Species: Human, Mouse; Application: WB, IP, IHC, ICC; Citation: Human NLRP1 is a sensor for double-stranded RNA. S. Bauernfried; Science (2021).; Website: https://adipogen.com/ag-25b-0006-anti-asc-pab-al177.html/
17. anti-NLRP3 (Adipogen, AG-20B-0014-C100, 1:200): Species: Human, Mouse; Application: WB, IP, ICC; Citation: Caspase-6 Is a Key Regulator of Innate Immunity, Inflammasome Activation, and Host Defense: Min Zheng; Cell (2020).; Website: https://adipogen.com/ag-20b-0010-anti-zbp1-mab-zippy-1.html
18. anti-NINJ1 (Customized): Species: Human, Mouse; Application: WB, IP, ICC; Citation: Ninjurin1 positively regulates osteoclast development by enhancing the survival of prefusion osteoclasts: Bae, S.J. Exp Mol Med (2019)
19. anti-LDHA (Proteintech, 19987-1-AP): Species: Human, Mouse, Rat; Application: WB, IP, IHC, ICC; Citation: RIP3 targets pyruvate dehydrogenase complex to increase aerobic respiration in TNF-induced necroptosis: Zhentao Yang; Nat Cell Biol (2018).; Website: https://www.ptglab.com/products/LDHA-Specific-Antibody-19987-1-AP.htm
20. anti-HMGB1 (Abcam, ab79823): Species: Human, Mouse, Rat; Application: WB, IHC, ICC; Citation: HMGB1 orchestrates STING-mediated senescence via TRIM30α modulation in cancer cells: Je-Jung Lee; Website: https://www.abcam.com/hmgb1-antibody-epr3507-ab79823.html
21. anti-cleaved IL-1β (CST, #63124): Species: Mouse; Application: WB, IP; Citation: Phenotype-based screening rediscovered benzopyran-embedded microtubule inhibitors as anti-neuroinflammatory agents by modulating the tubulin-p65 interaction: Junhyeong Yim; https://www.cellsignal.com/products/primary-antibodies/cleaved-il-1b-asp117-e7v2a-rabbit-mab-mouse-specific/63124
22. anti-IL-1β (CST, #12426): Species: Mouse; Application: WB; Citation: A highly conserved host lipase deacylates oxidized phospholipids and ameliorates acute lung injury in mice: Benkun Zou; Website: https://www.cellsignal.com/products/primary-antibodies/il-1b-d4t2d-rabbit-mab-mouse-specific/12426
23. anti-IL-18 (Abcam, ab207323): Species: Mouse; Application: WB, IP; Citation: Salidroside ameliorates Parkinson's disease by inhibiting NLRP3-dependent pyroptosis: Zue Zhang; Website: https://www.abcam.com/il-18-antibody-epr19956-ab207323.html
24. anti-GAPDH (Santacruz, sc-166574 HRP): Species: Human, Mouse, Rat; Application: WB, IP, IF, IHC; Citation: TAZ links exercise to mitochondrial biogenesis via mitochondrial transcription factor A: Jun-Ha Hwang; Website: https://www.scbt.com/p/gapdh-antibody-h-12
25. anti-β-actin (Proteintech, 66009-1-IG): Species: Human, Mouse, Rat; Application: WB, IP, IF, IHC; Citation: Caspase-6 Is a Key Regulator of Innate Immunity, Inflammasome Activation, and Host Defense: Min Zheng; Cell (2020); Website: https://www.ptglab.com/products/Pan-Actin-Antibody-66009-1-Ig.htm
26. anti-IgG (CST, #3900): Application: IP, IF, IHC; Citation: FGF18 alleviates hepatic ischemia-reperfusion injury via the USP16-mediated KEAP1/Nrf2 signaling pathway in male mice.; https://www.cellsignal.com/products/primary-antibodies/rabbit-da1e-mab-igg-xp-isotype-control/3900

Eukaryotic cell lines

Policy information about cell lines and Sex and Gender in Research

Cell line source(s)	THP-1: ATCC® TIB-202™, RAW264.7: ATCC®TIB-71™, L929: ATCC®CCL-1™
Authentication	The THP-1, RAW264.7 and L929 cell lines were purchased directly from ATCC and were not further authenticated in our lab.
Mycoplasma contamination	Cells were tested for mycoplasma contamination using mycoplasma detection PCR and were found to be negative for mycoplasma contamination.
Commonly misidentified lines (See ICLAC register)	Commonly misidentified lines have not been used in this study.

Animals and other research organisms

Policy information about studies involving animals; ARRIVE guidelines recommended for reporting animal research, and Sex and Gender in Research

Laboratory animals	WT C57/BL6 (J substrain) mice were originally obtained from Jackson Laboratory (000664) and then interbred in our colony at St. Jude. All genetically modified mouse lines were extensively backcrossed to our WT line. All mice were bred at the Animal Resources Center at St. Jude Children's Research Hospital under specific pathogen-free conditions. Both male and female mice were used in this study; age- and sex-matched 6- to 9-week-old mice were used for in vitro studies, and age- and sex-matched 6- to 8-week-old mice were used for in vivo studies. Mice were housed in 20-23.3 degrees Celsius and 30-70% humidity with 12 h light/dark cycles and were fed standard chow. During the heat shock in vivo model, mice were housed at 39 degrees Celsius for 2 h before being transferred back to standard housing conditions. Animal studies were conducted under protocols approved by the St. Jude Children's Research Hospital committee on the Use and Care of Animals (protocol 482).
Wild animals	The study did not involve wild animals.
Reporting on sex	Sex-matched mice were used for all experiments. We did not observe any notable differences in responses between males and females.

Field-collected samples	The study did not involve field collected samples.
Ethics oversight	All mice were bred at St. Jude. Animal studies were conducted in accordance with protocols approved by the St. Jude Animal Care and Use Committee (protocol 482).

Note that full information on the approval of the study protocol must also be provided in the manuscript.
