# Supplementary material for: NINJ1 mediates inflammatory cell death, PANoptosis, and lethality during infection conditions and heat stress
Source: Nat Commun. 2024 Feb 26;15:1739. doi: 10.1038/s41467-024-45466-x (PMC10897308; doi:10.1038/s41467-024-45466-x)
Supplement: Supplementary file 7 — Source Data [file 41467_2024_45466_MOESM7_ESM.zip › Final Source Data Files/Uncropped WB.pdf]

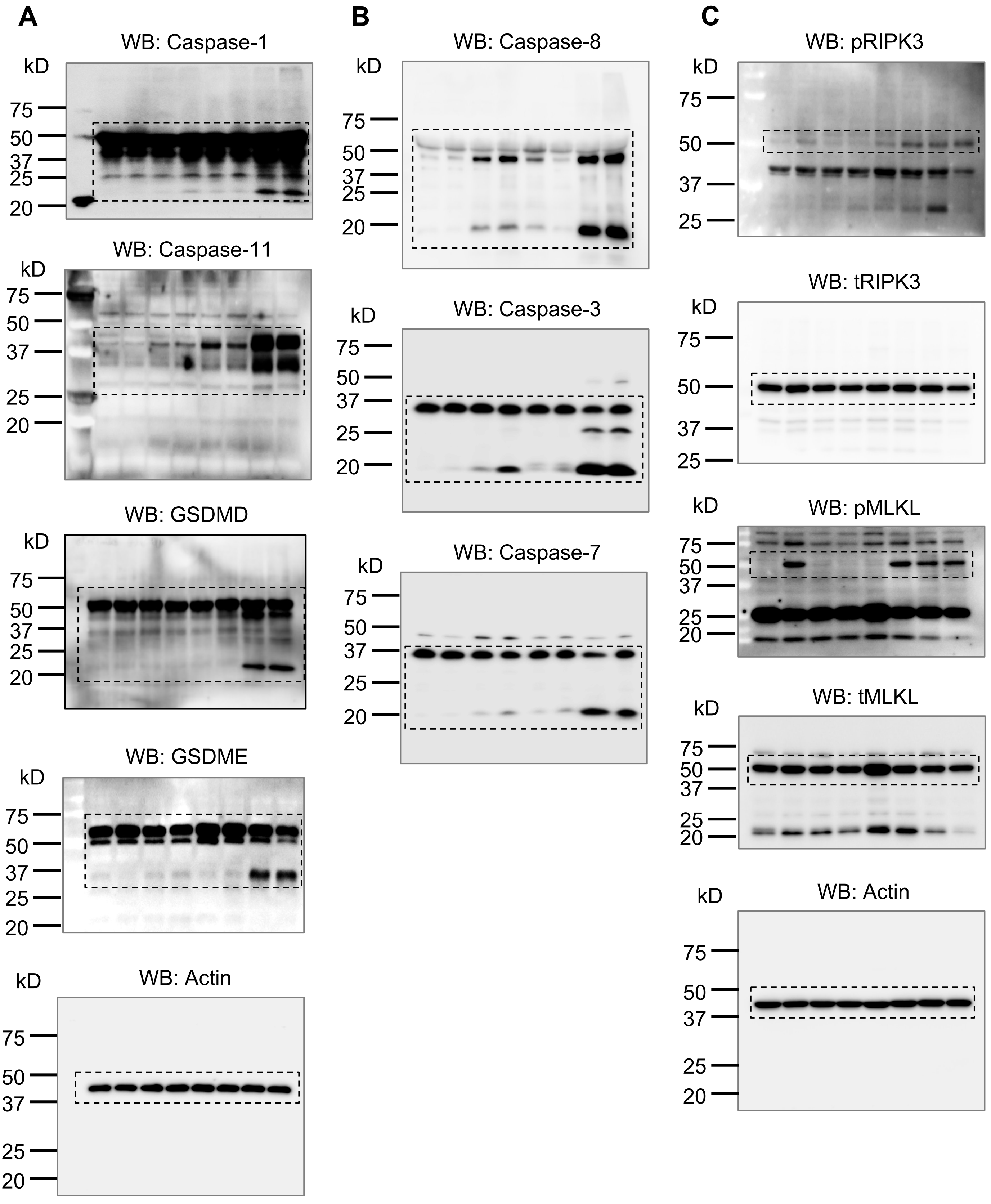
A
WB: Caspase-1
kD
75
50
37
25
20
WB: Caspase-11
WB: GSDMD
WB: GSDME
WB: Actin
B
WB: Caspase-8
WB: Caspase-3
WB: Caspase-7
C
WB: pRIPK3
WB: tRIPK3
WB: pMLKL
WB: tMLKL
WB: Actin

Figure 2

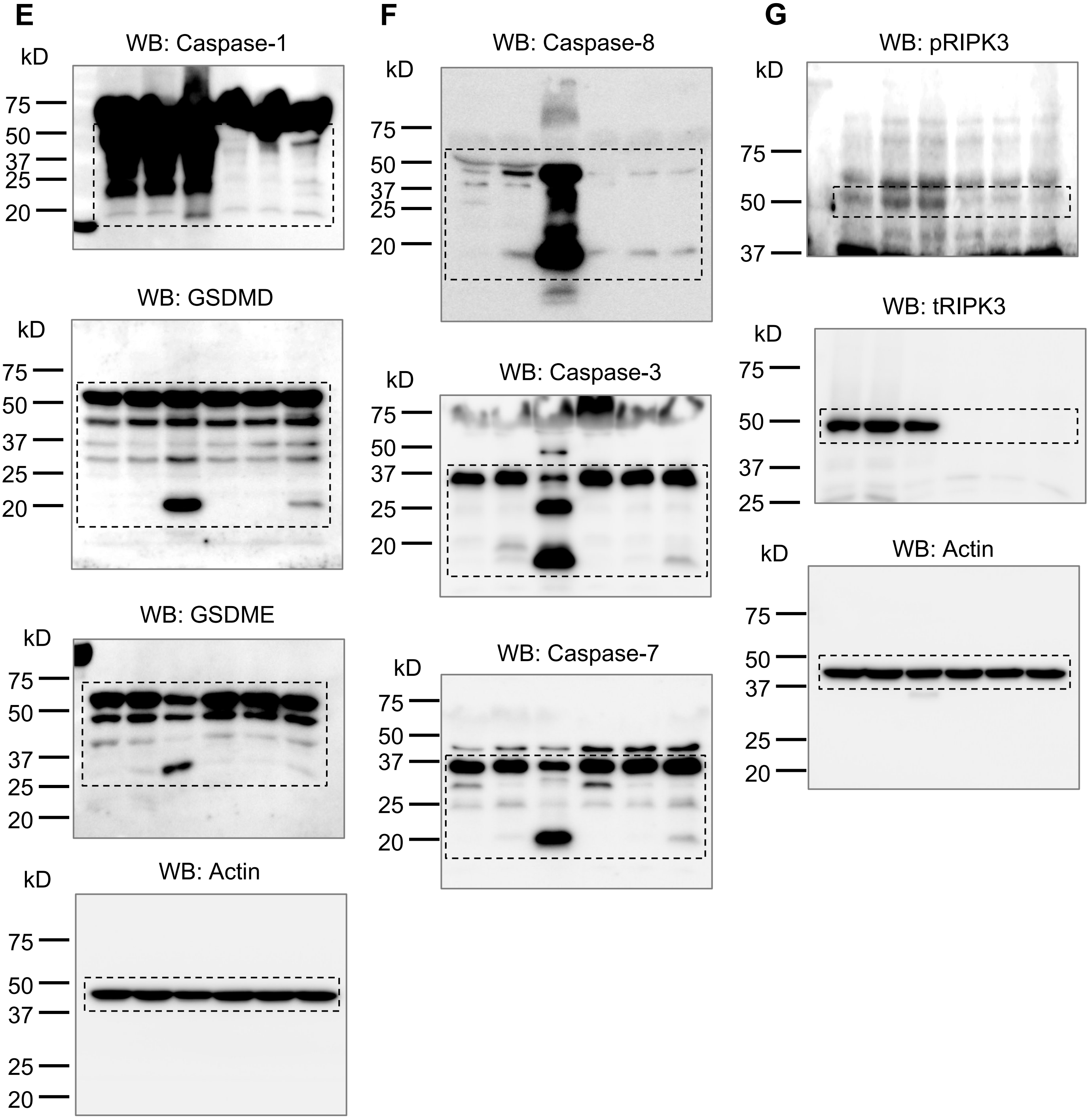
E
WB: Caspase-1
kD
75
50
37
25
20
WB: GSDMD
kD
75
50
37
25
20
WB: GSDME
kD
75
50
37
25
20
WB: Actin
kD
75
50
37
25
20
F
WB: Caspase-8
kD
75
50
37
25
20
WB: Caspase-3
kD
75
50
37
25
20
WB: Caspase-7
kD
75
50
37
25
20
G
WB: pRIPK3
kD
75
50
37
WB: tRIPK3
kD
75
50
37
25
WB: Actin
kD
75
50
37
25
20

Figure 2

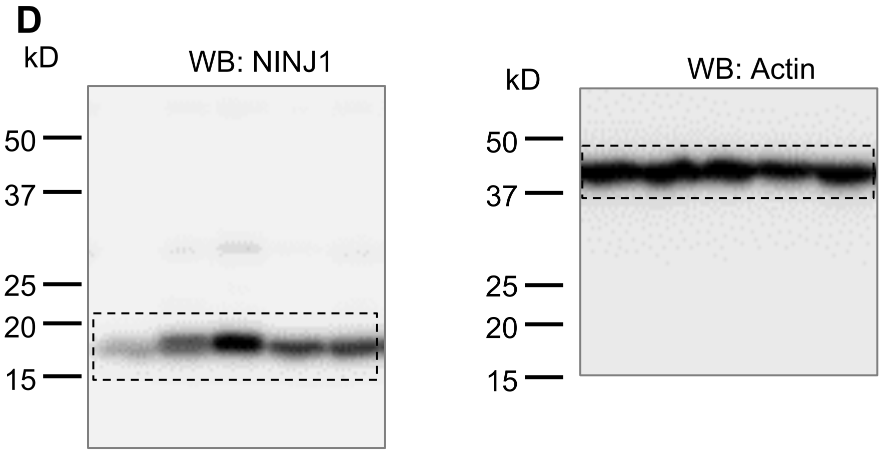

Figure 4

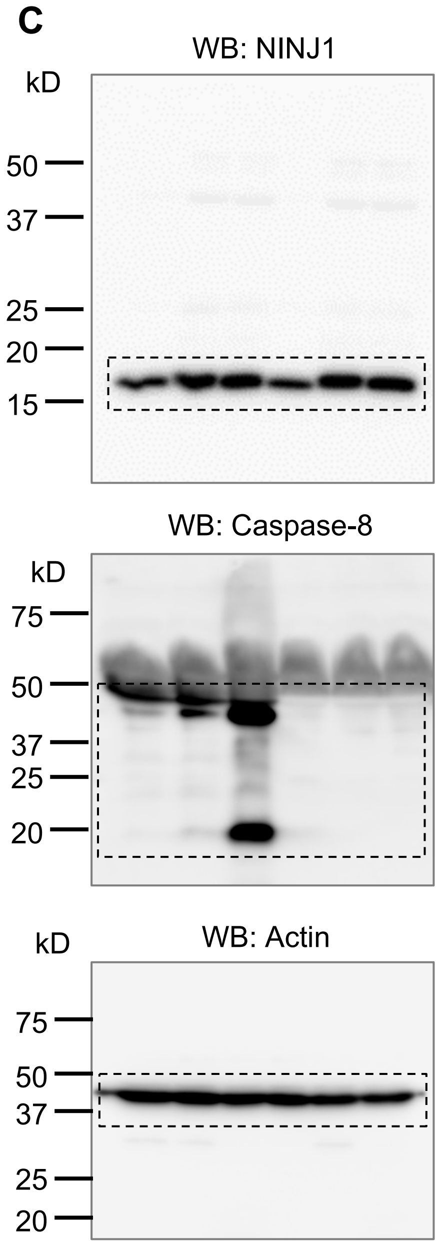
C
WB: NINJ1
kD
50
37
25
20
15
WB: Caspase-8
kD
75
50
37
25
20
WB: Actin
kD
75
50
37
25
20

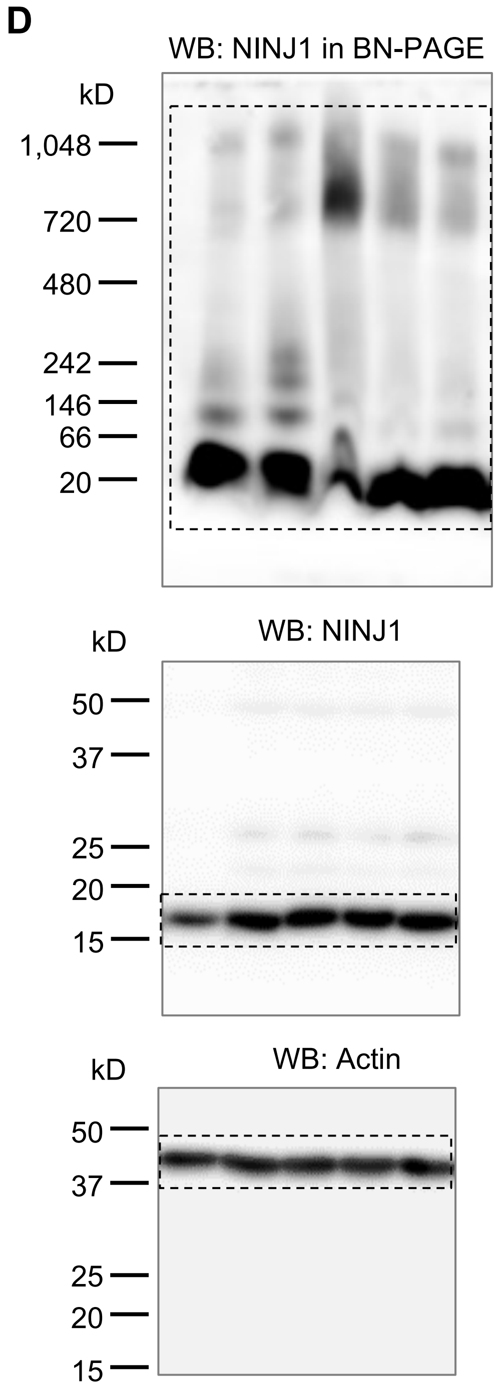
D
WB: NINJ1 in BN-PAGE
kD
1,048
720
480
242
146
66
20
WB: NINJ1
kD
50
37
25
20
15
WB: Actin
kD
50
37
25
20
15

Figure 5

A

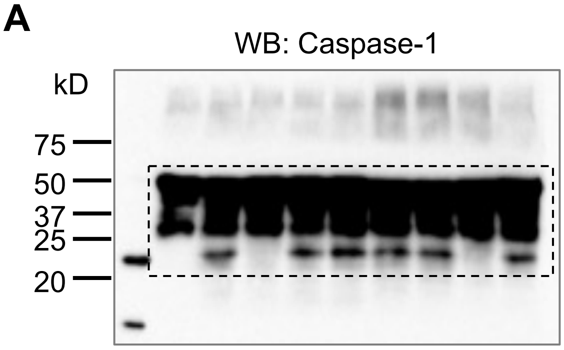

Supplementary Figure 3

A

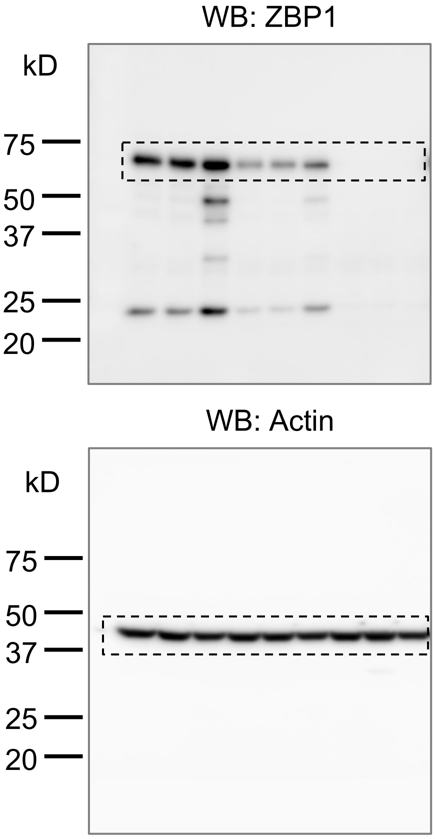

C

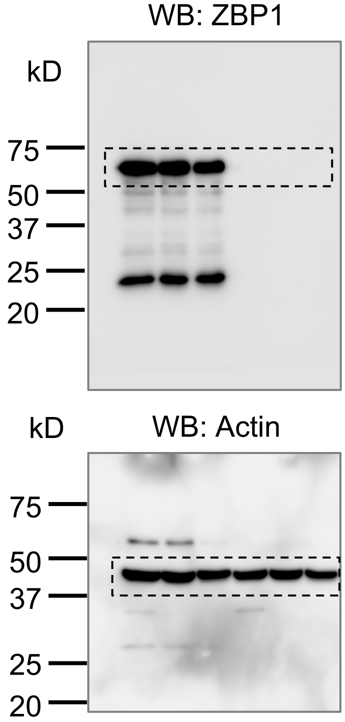

Supplementary Figure 4

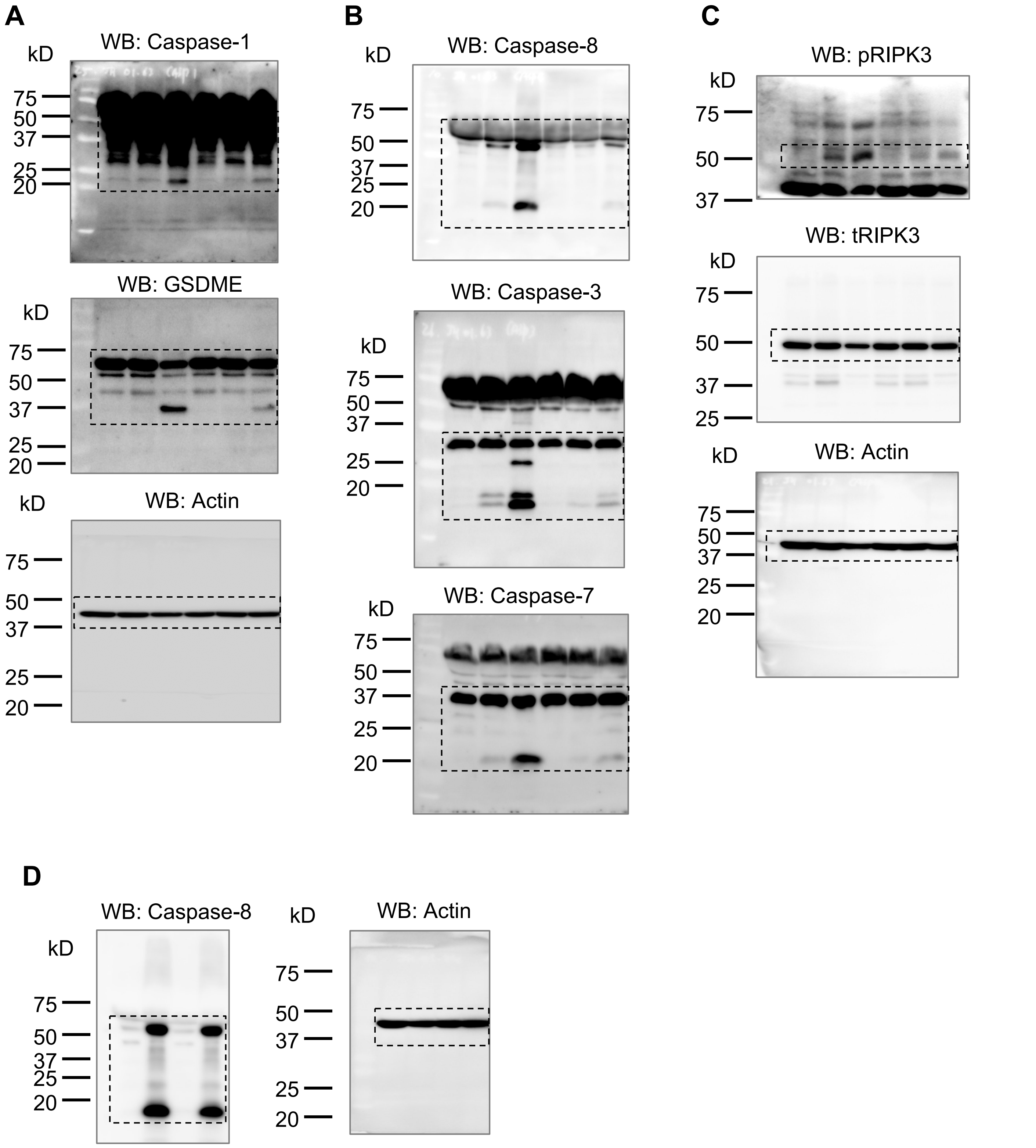

Supplementary Figure 5

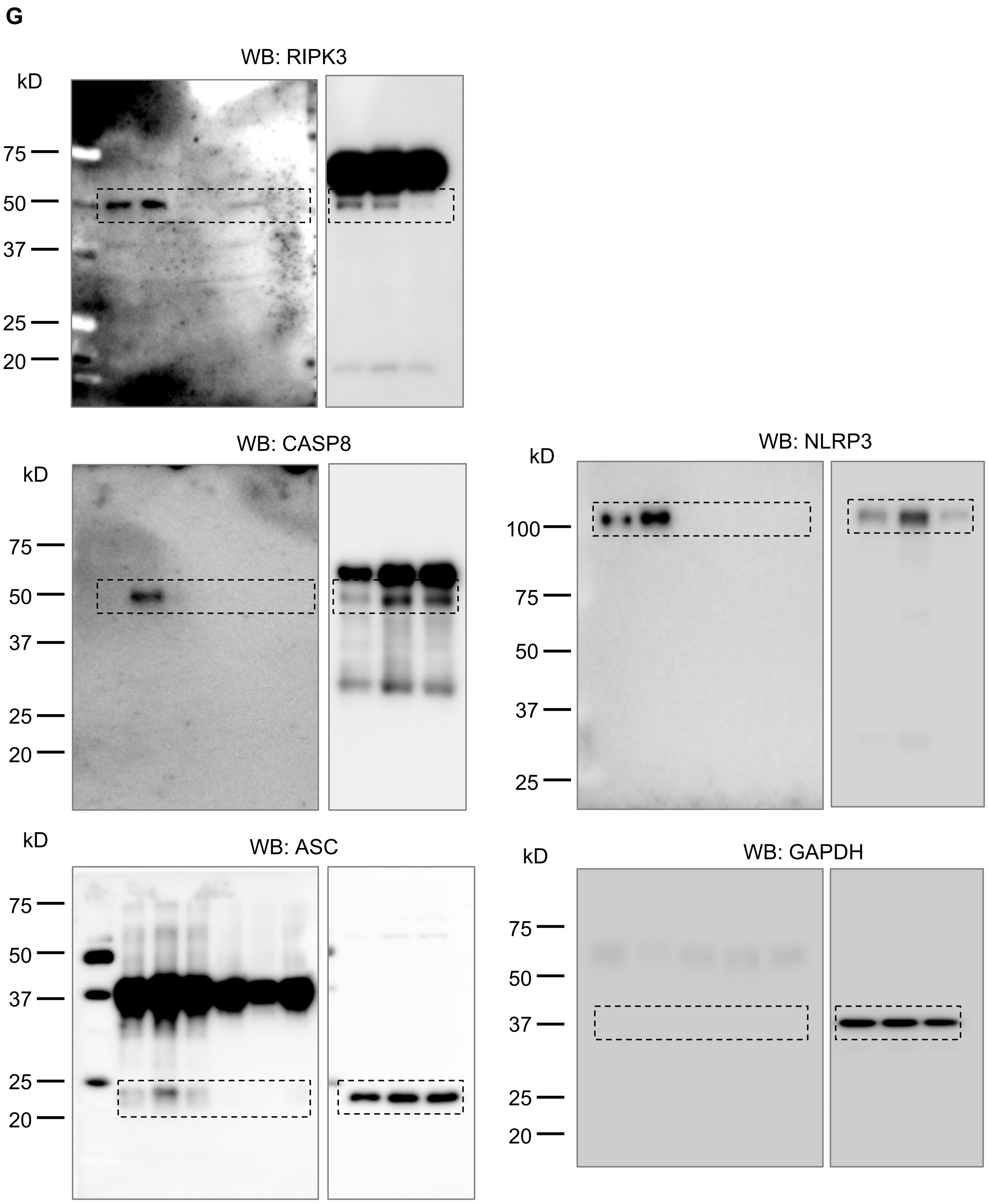

Supplementary Figure 6

B

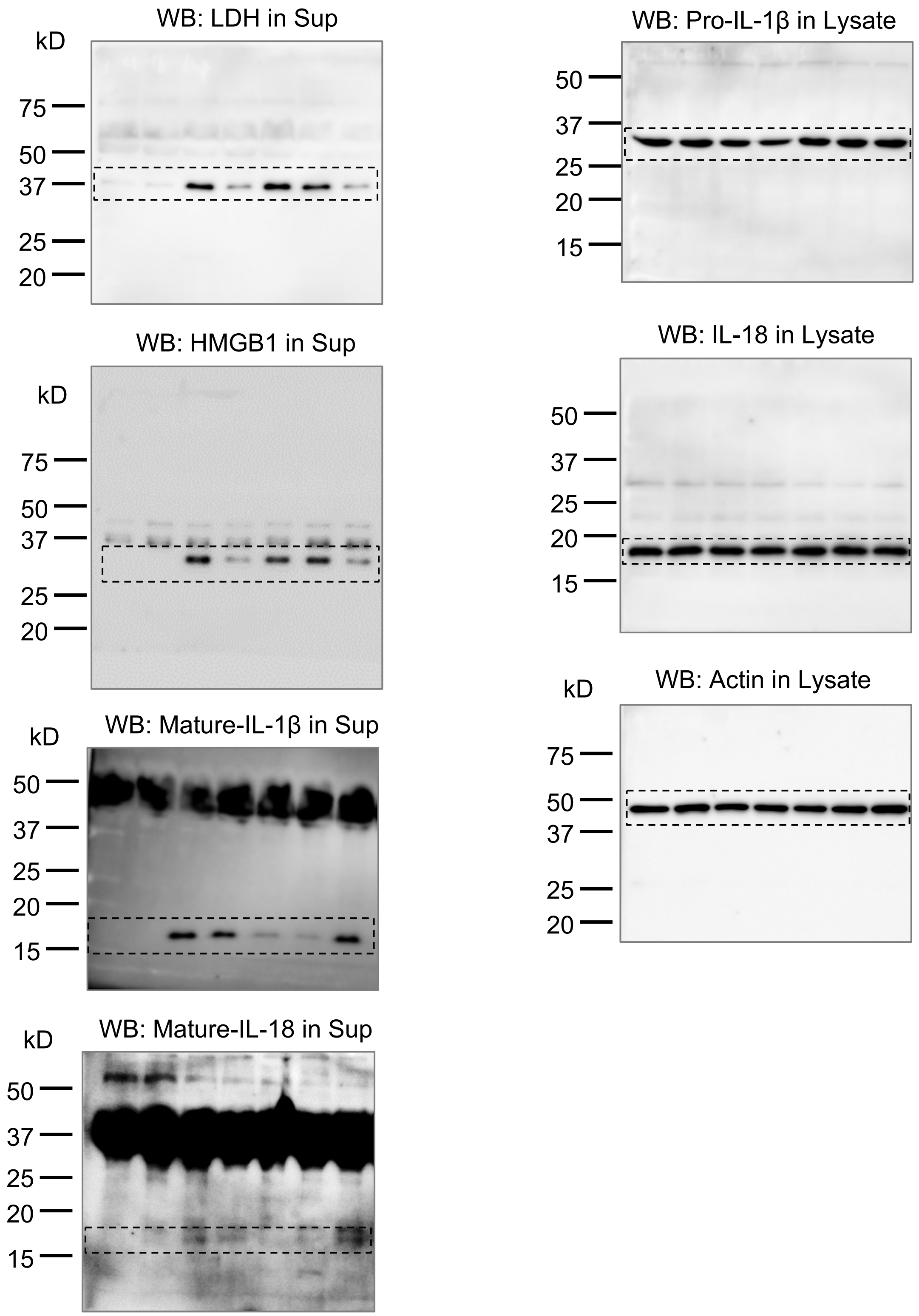

Supplementary Figure 8

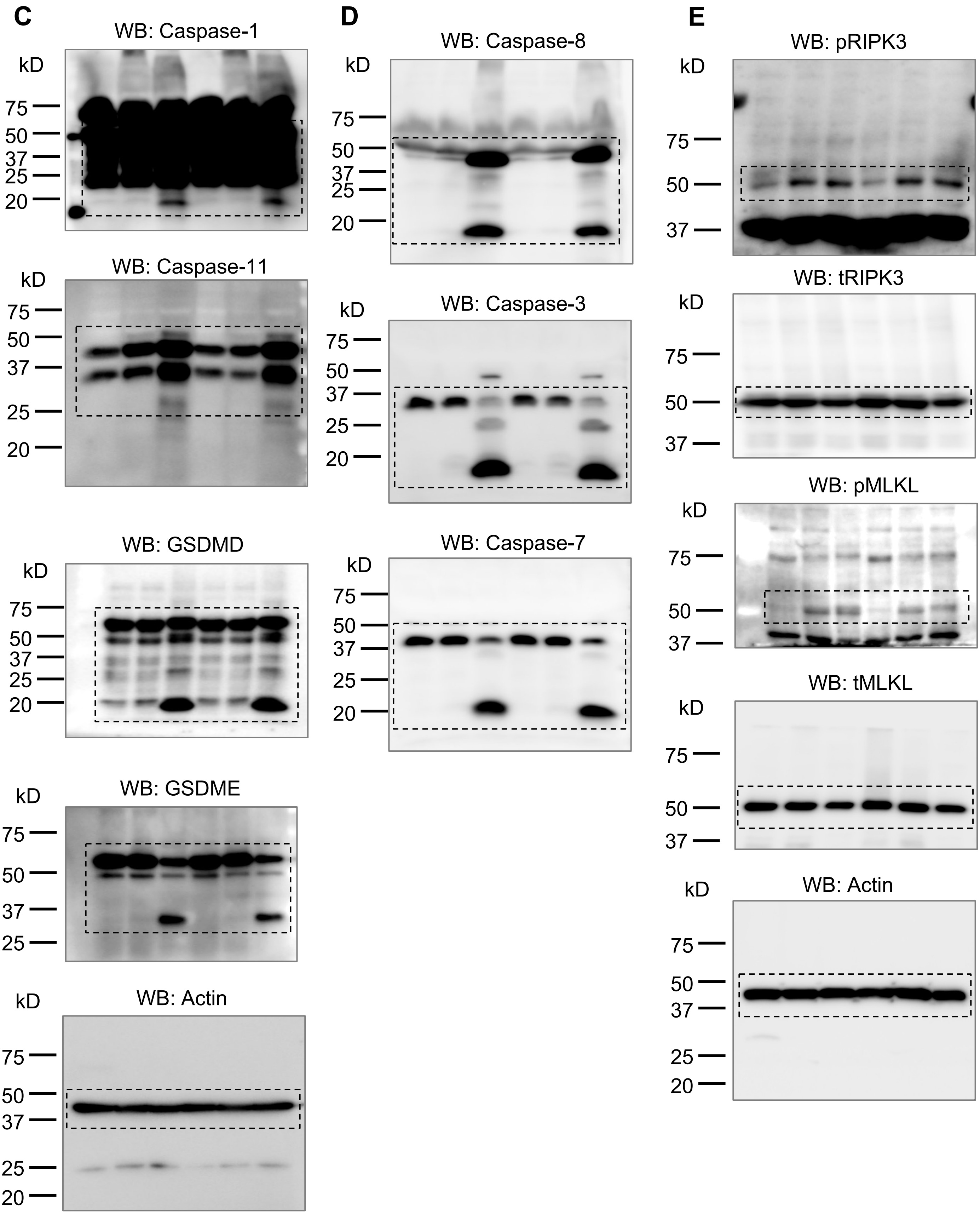

Supplementary Figure 8
